# The Improved Hybrid STD– Radial Basis Function Neural Network Approach for Time Series Forecasting Application to Tesla Stock Price Prediction

**DOI:** 10.12688/f1000research.172498.1

**Published:** 2026-02-18

**Authors:** Hiba H. Abdullah, Nooruldeen A. Noori, Taha S. Hamza

**Affiliations:** 1 mathematics, Tikrit University, Tikrit, Saladin Governorate, 34001, Iraq; 2 mathematics, University of Fallujah, Al-Fallujah, Al Anbar Governorate, 31002, Iraq

**Keywords:** Time series decomposition, Stock price forecasting, RBFNN, Hybrid neural network, STD, and Tesla stock prediction

## Abstract

The forecast of time series in financial applications is difficult to perform as time series forecasting is nonlinear in nature, seasonal, and has structural variability. Stock price series tend to follow a lot of nonlinear dynamics, which undermines the power of single-model approaches. Hybrid decomposition-based models have attracted increasing interest in order to gain accuracy by separating heterogeneous features from one another. In this work, we present a hybrid forecasting methodology that incorporates STD decomposition with RBFNN (Radial Basis Function Neural Network). The time series is decomposed, where trend, seasonal, and dispersion components are separately modeled using RBFNN with Gaussian basis functions. The predicted feature sets are then recombined to construct a forecast, to be evaluated with weekly Tesla stock price data and standard accuracy performance metrics. The STD–RBFNN model gives very low forecasting errors under different variables and a high coefficient of determination. It shows superiority compared with an alternative hybrid neural network model, especially in modeling nonlinear variation under similar experimental conditions. This results in substantially greater forecasting accuracy because trend, seasonal, and dispersion components separate before neural modeling. The proposed STD and RBFNN pipeline is a good and highly flexible method to forecast complex nonlinear and seasonal financial time series.

## Introduction

Time series forecasting poses a fundamental challenge in the fields of economics, energy, and finance, particularly when the data exhibits complex, nonlinear, seasonal, and structural characteristics. One of the most common and essential forecasting applications is predicting stock prices, which are characterized by volatility, instability, and significant exposure to external factors, making traditional models often inadequate for accurately representing them.

In this paper, the hybrid models that have emerged with the blend of traditional analytical methods and sophisticated machine learning methods are important. One of these tools is Trend and Seasonality decomposition (STD) which enables the decomposition of the time series into three main components of trend, seasonality and dispersion. This decomposition aids in grasping the internal framework of the series which makes it possible to apply forecasting methods appropriate for each component. On the other hand, Radial Basis Function Neural Networks (RBFNNs) provide an efficient solution to intricate issues by partitioning the learning into independent subnetworks. Each subnetwork specializes in learning a distinct pattern or component of the data. Afterward, the subnetwork outputs are integrated to form a consolidated predictive decision. This method is very effective in resolving the dimensionality problem, enhancing the generalization ability of the model, and decreasing the chances of overlearning.

Previous investigation into hybrid models has been conducted. As an example, Zhang (2003) used combination of linear models and neural networks in the time series forecasting. ^1^ On the other hand, Hyndman et al. (2008) with the use of STL and other seasonal decomposition models focused on pattern analytics. ^2^ In recent years, models based on LSTM and GRU have also been created for the forecasting of nonlinear time series. However, to the best of my knowledge, no effort has been made on the systematic integration of the STD model with the RBFNN architecture. The synthesis of components into a whole has been the focus of numerous studies. ^3^ In 2020, a research proposed a hybrid model for metro ridership forecasting which integrates trend and seasonal decomposition using LOESS and LSTM for short-term forecasting. Several hybrid approaches to analysis and neural networks been developed in the scientific literature as STDR-MNN by Aljboori, in 2023 combined dispersion analysis with standard neural networks, ^4^ STL-FNN by Sultana, and Aljbooria, in 2024, implemented seasonal trend analysis using feedforward neural network, ^5^ STR-ENN by Othman, and Aljboori, in 2025, presented advanced neural architectures with regression-based analysis. ^6^ However, no previous studies systematically integrates STD analysis with RBFNNs. This represents a gap the scientific literature and justifies the novelty of this study.

Based on this background, this study aims to develop a hybrid predictive model that combines STD analysis and RBFNN technology. Each component of the series is assigned to a specialized neural unit within the standard network, trained and modeled separately, and then combined to predict the entire time series. This model was applied to Tesla stock price data to verify its predictive effectiveness and efficiency.

## Decompose trend, seasonality, and dispersion using regression (STD)

This is an evolutionary model of the Decompose Trend, Seasonality, and Remainder (STR) model. The simplest STD model describes a time series $x_{t}$ consisting of three components as in the equation below ^7^:

$$x_{t}=T_{t}+S_{t}\times D_{t}$$ (1)

where $T_{t}$ is the cyclic trend, $S_{t}$ is the additional seasonal component, $D_{t}$ is the Dispersion component and t∈{1,2,…,n} and we have k as the moving average. Let $s_{n}\left(t\right)\;\in \;\left\{1,\ldots ,k\right\}$ which converts time t to the corresponding season $s_{n}\left(t\right)$ .

Take seasonal component $S_{t}$ with constant recurring pattern at time t . The seasonal pattern can be treated as a two-dimensional model, ${\left\{S_{i,t}\right\}}_{i=1}^{k}$ , and assume that $S_{t}=S_{s_{n}\left(t\right),t}$ (where S is a vector of seasons with a single index and S is a matrix of seasonal shapes in Equation (1). Thus, rewritten as form:

$$x_{t}=T_{t}+S_{s_{n}\left(t\right),t}\times D_{t}$$ (2)

where $S=\left[S_{s,t}\right]$ is a k×n matrix, where k represents the moving average and n represents the length of the time series.

This representation allows for simple constraints on the seasonal patterns represented by the matrix $S=\left[S_{s,t}\right]$ . The entire model can be described as follows ^8^:

•
$D_{t}$ represents the dispersion, which is $D_{t}\sim \mathrm{iid}\;N\left(0,\sigma_{I}^{2}\right)$ .
•
$T_{t}$ represents the smoothed trend under the conditions ${∆}^{2}T_{t}=T_{t+1}-2T_{t}+T_{t-1}$ such that $T_{t}\sim \mathrm{iid}\;N\left(0,\sigma_{T}^{2}\right)$ .
• The property $\sum_{s}S_{s,t}=0$ , where $S_{s,t}$ is the seasonal coefficients for any time t. Each seasonal term varies smoothly over time, the vector ${\left\{{∆}_{t}^{2}S_{s,t}\right\}}_{s=1}^{k}={\left\{S_{s,t+1}-2S_{s,t}+S_{s,t-1}\right\}}_{s=1}^{k}$ is inside the vector $S_{s,t}\sim \mathrm{iid}\;N\left(0,\sigma_{S}^{2}\Sigma_{S}\right),\forall t$ , where $\Sigma_{S}$ is matrix of dimension k×k and considered the covariance matrix of k random variables $\xi_{s}=\lambda_{s}+\frac{1}{k}\sum_{r=1}^{k}\lambda_{s}$ obtained from $\lambda_{1},\ldots ,\lambda_{k}\sim \mathrm{iid}\;N\left(0,1\right)$ .
• The model parameters are given by $\sigma_{I}^{2}$ , $\sigma_{T}^{2}$ , $T_{0}$ , $T_{1}$ , $S_{s,0}$ , $S_{s,1}$ , or ${\left\{S_{s,n}\right\}}_{s=1}^{k}$ .

Let $x_{t}$ be any time series, then the components for it can be found by putting it in the form ${\left\{{\left\{x_{i,j}\right\}}_{j=1}^{m}\right\}}_{i=1}^{K}=\left\{{\left\{x_{1,j}\right\}}_{j=1}^{m},\ldots ,{\left\{x_{K,j}\right\}}_{j=1}^{m}\right\}$ , where m is the number of periods and $K=\frac{n}{m}$ , K∈N , i=1,2,…,K , j=1,2,…,m is the time index inside the given seasonal cycle and global index calculated by t=m(i+1)+j , then the, as follows:

$${\overset{̿}{x}}_{i}=\frac{1}{m}\sum_{j=1}^{m}x_{i,j}$$ (3)

Hence

$${\left\{T_{t}\right\}}_{t=1}^{n}={\left\{\underset{n-\text{times}}{\underbrace{\left\{{\overset{̿}{x}}_{i},\ldots ,{\overset{̿}{x}}_{i}\right\}}}\right\}}_{i=1}^{K}$$ (4)

Its diversity measure is defined as follows:

$${\tilde{x}}_{t}=\sqrt{\sum_{j=1}^{m}{\left(x_{i,j}-{\overset{̿}{x}}_{i}\right)}^{2}}$$ (5)

While the dispersion component is defined using diversities from Equation (5) of these sequences:

$${\left\{D_{t}\right\}}_{t=1}^{n}={\left\{\underset{n-\text{times}}{\underbrace{\left\{{\tilde{x}}_{i},\ldots ,{\tilde{x}}_{i}\right\}}}\right\}}_{i=1}^{K}$$ (6)

Based on the Trend component in Equation (4) and the Dispersion in Equation (6), the Seasonal component can be obtained by the following equation:

$$S_{t}=\frac{x_{t}-T_{t}}{D_{t}}$$ (7)

In this case, we assume that our time series $x_{t}$ can be written in terms of autoregressive moving averages as follows ^9, 10^:

$$x_{t}=T_{t}+\left(\sum_{i=1}^{I}S_{t}^{i}+\sum_{i=1}^{p}\alpha_{p,i}y_{t-i}+\sum_{j=1}^{q}\beta_{j}z_{q,t-j}\right)\times D_{t}$$ (8)

where $z_{q,t}$ represents the covariates with coefficients $\alpha_{p,t}$ , which \ be time-varying and even seasonal.

**Figure 1.**
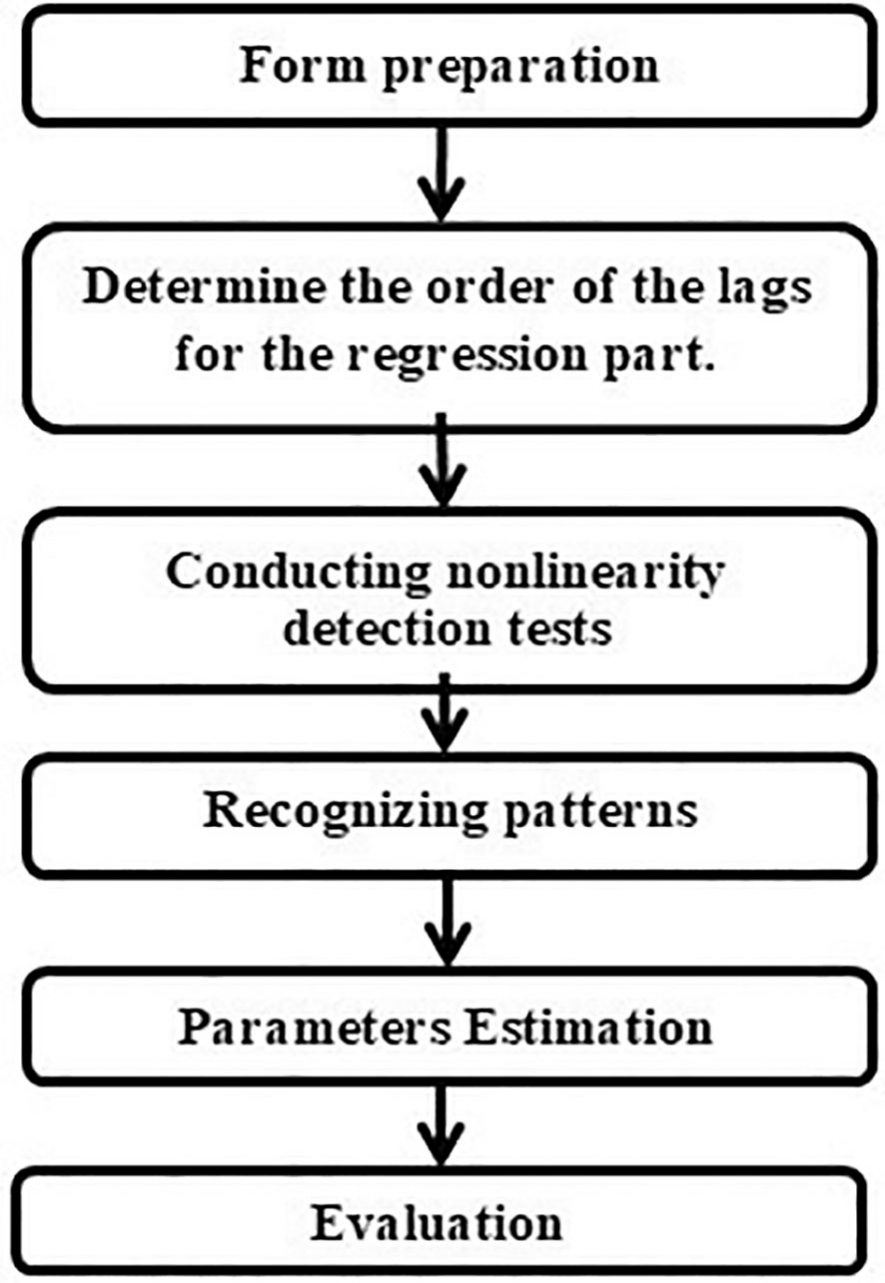
STD modeling process flowchart.

The modeling process for an STD model roughly involves three steps: model development, parameter estimation, and model evaluation. The model development process consists of determining the lags of the regression component, testing for nonlinearity, and identifying patterns. The model evaluation phase includes goodness-of-fit testing and fitness testing. Figure 1 displays a flowchart of the modeling steps for the STD model domains.

## Radial basis function neural network (RBFNN)

A RBFNN is a type of artificial neural network used in machine learning and data processing. RBFNN consists of three main layers: the first layer is the input layer, followed by an intermediate layer called the hidden layer, which contains units known as Radial Basis Functions (RBFs) are a mathematical function based on the Euclidean distance between the input point and the centers of the functions to measure the impact of each RBF on the final result, and finally the output layer. Each hidden unit applies an RBF, most commonly the Gaussian function, which is defined as:

$$\Phi_{i}\left(x\right)=\exp\left(-\frac{{\left\|x-c_{i}\right\|}^{2}}{2\sigma_{i}^{2}}\right)$$ (9)

where x is the input vector, $c_{i}$ the center of $i^{\mathrm{th}}$ RFB unit, and $\sigma_{i}$ the spread (width) of the function.

The output is computed as a weighted sum of activations of hidden units:

$$y\left(x\right)=\sum_{i=1}^{N}w_{i}\Phi_{i}\left(x\right)+b$$ (10)

where $w_{i}$ are connection weights from hidden units to the output layer, and b is a bias term.

RBFNN is typically trained in two stages ^11^:

i. Determining the centers $c_{i}$ and $\sigma_{i}$ , often using clustering algorithms such as K -mean
ii. Estimating output weights using linear regression or pseudo-inverse methods:
$$W=\Omega^{T}Y$$ (11)

where Ω is a matrix of RBF activations for training inputs and Y is the corresponding target output vector.

To minimize the prediction error, a cast function such as the mean squared error (MSE) can be used. ^12, 13^

Application of an RBFNN

Implementing the RBFNN neural network involves several key steps. Here are the most critical steps that can be followed ^14, 15^:

1. Identifying the network architecture: This involves defining the layers and the number of components each layer will have. For instance, in an RBFNN, there would be an input layer for the variables, an intermediate layer containing the RBFs, and an output layer that contains the predicted output.
2. Information gathering and preparation: The RBFNN model necessitates RBF input data for both training and validation. This input data requires preprocessing, so the network will be able to use the data, and also split into training and validation subsets.
3. Locating the RBF centers: You have to locate the centers of RBF which are positions in multidimensional space that are RBF centers. These centers can be found using K -means algorithms or Mahalanobis distance.
4. Determine assigned RBF weight: Every RBF Center should be assigned appropriate weight which in turn should be adjustable according to each RBF. The effect of each RBF on the network score will be influenced by these weights.
5. Training the Network: In this stage, the prepared data, now split into a training set and a test set, is utilized to train the network. The objective is to modify the weights and centers of the RBF so that the network achieves the desired accuracy in predicting the training data.
6. Evaluate performance using the test: After the network has been trained, it must be evaluated using a separate test data set. This evaluation is important because it helps us understand how well the network has been trained and, most importantly, how well it is able to generalize to new, unseen data.
7. Adjusting weigh and center goal: performance optimization: Improvement is possible with the RBFNN through increasing the weights and modifying the centers of the RBF although this is highly dependent on a clear understanding of the effect different functions have on the network’s results.
8. Implementation of trained network (Deployment): Once the network has been trained and its accuracy tuned, it is now ready to be placed into the production environment where it can make predictions on streaming new data.

By following these steps, the RBFNN can be successfully applied and implemented for a wide range of problems in machine learning and prediction. ^16^

## The improved hybrid STD–radial basis function neural network (STD-RBFNN)

The STD-RBFNN model is a hybrid forecasting framework that combines time series analysis using STD and RBFNN. This model first decomposes the time series into its principal components: trend, seasonality, dispersion, and remainder, if necessary. This method uses the STD technique to accurately separate nonlinear patterns. The RBFNN is then trained independently on each of these components to learn and effectively represent nonlinear relationships. After training, the model predicts each component separately, and these predicted components are then recombined to obtain the final forecast of the original time series. This approach has proven effective in improving forecasting accuracy on complex data, as it treats each component of the series separately and leverages the RBFNN's ability to capture subtle nonlinear patterns.

If we have a data series with a vector $X=\left\langle x_{1},x_{2},\ldots ,x_{n}\right\rangle$ , n is the number of observations (inputs) and we want to predict h of future steps $\hat{X}=\left\langle {\hat{x}}_{n+1},{\hat{x}}_{n+2},\ldots ,{\hat{x}}_{n+h}\right\rangle$ (outputs), Then the prediction steps for the improved hybrid model in detail are as follows:

**Step 1:** Input the data sets.

**Step 2:** Splitting the data series into two series, the training series and the prediction test series.

**Step 3:** STD Analysis The training series is decomposed into three components: trend, seasonality, and dispersion.

**Step 4:** The data for each component $Z_{t}\;\in \;\left\{T_{t},S_{t},D_{t}\right\}$ is divided into a training set $Z^{\text{train}}$ and a test set $Z^{\text{test}}$ and the future prediction step h is determined.

**Step 5:** RBFNN Neural Network Training An independent RBF neural network is trained for each of the three components, using the Gaussian radial basis function from Equation (9). The network output is represented by modifying (10) as:

$${\hat{Z}}_{t}=\sum_{i=1}^{N}w_{i}\Phi_{i}\left(x\right)+b$$ (12)

And then the weights are calculated using the pseudo-inverse by modifying Equation (11) to the form:

$$W=\Omega^{T}Z^{\text{train}}$$ (13)

Additionally, the number of nodes is determined based on the target, the width of the RBF function, and the threshold used in the RBFNN.

**Step 6:** Combine the outputs to predict the original series for all points in the test set by modifying Equation (1) as follows:

$${\hat{x}}_{t}={\hat{T}}_{t}+{\hat{S}}_{t}\times {\hat{D}}_{t}$$ (14)

**Step 7:** The accuracy of the model is evaluated using two indicators, the mean square error (MSE), the root mean square error (RMSE), Mean Absolute Error (MAE), Mean Absolute Percentage Error (MAPE), and Coefficient of Determination ( $R^{2}$ ), defined respectively by the equations ^16– 18^:

$$\mathrm{MSE}=\frac{1}{n}\sum_{i=1}^{n}{\left(x_{i}-{\hat{x}}_{i}\right)}^{2}$$ (15)

$$\text{RMSE}=\sqrt{\frac{1}{n}\sum_{i=1}^{n}{\left(x_{i}-{\hat{x}}_{i}\right)}^{2}}$$ (16)

$$\mathrm{MAE}=\frac{1}{n}\sum_{i=1}^{n}\left|x_{i}-{\hat{x}}_{i}\right|$$ (17)

$$\text{MAPE}=\frac{100\%}{n}\sum_{i=1}^{n}\left|\frac{x_{i}-{\hat{x}}_{i}}{x_{i}}\right|$$ (18)

$$R^{2}=1-\frac{\sum_{i=1}^{n}\left|x_{i}-{\hat{x}}_{i}\right|}{\sum_{i=1}^{n}\left|x_{i}-\bar{x}\right|}$$ (19)

where $x_{i}$ , ${\hat{x}}_{i}$ and $\bar{x}$ are the real value, predicted value and Average real value, respectively, and n is the time series length.

**Step 8:** Optimize the model and parameters if the prediction results are not acceptable. Adjust the number of nodes, the width of the RBF function, and the threshold used in RBFNN again. Then modify the analysis windows in STD, such as the seasonal period and trend range. Re-evaluate using MAE and RMSE and choose the setting that gives the best performance on the test set.

**Step 9:** Final prediction. After determining the best setting, the model is retrained using the complete data and is used to predict h future values:

$${\hat{x}}_{n+1},{\hat{x}}_{n+2},\ldots ,{\hat{x}}_{n+h}$$

**Figure 2.**
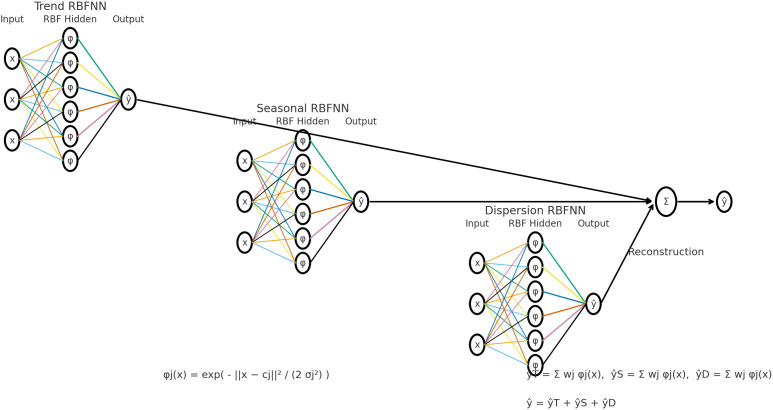
Hybrid method diagram.

Figure 2 displays the diagram illustrates the steps for implementing the hybrid model.

## Efficiency analysis and performance evaluation of the STL-RBFNN hybrid model using real-world data (stock prices)

In this section, the efficiency of the proposed hybrid STL-RBFNN model is evaluated by applying it to real data representing Tesla stock prices from October 23, 2022, to July 20, 2025, collected from the global website https://www.investing.com/equities/tesla-motors-historical-data . These data are as follows:

228.52 207.47 195.97 180.19 182.86 194.86 179.05 150.23 123.15 123.18 113.06 122.40 133.42 177.90 189.98 196.89 208.31 196.88 197.79 173.44 180.13 190.41 207.46 185.06 185.00 165.08 164.31 170.06 167.98 180.14 193.17 213.97 244.40 260.54 256.60 261.77 274.43 281.38 260.02 266.44 253.86 242.65 215.49 238.59 245.01 248.50 274.39 244.88 250.22 260.53 251.12 211.99 207.30 219.96 214.65 234.30 235.45 238.83 243.84 253.50 252.54 248.48 237.49 218.89 212.19 183.25 187.91 193.57 199.95 191.97 202.64 175.34 163.57 170.83 175.79 164.90 171.05 147.05 168.29 181.19 168.47 177.46 179.24 178.08 177.48 178.01 183.01 197.88 251.52 248.23 239.20 219.80 207.67 200.00 216.12 220.32 214.11 210.73 230.29 238.25 260.46 250.08 217.80 220.70 269.19 248.98 321.22 320.72 352.56 345.16 389.22 436.23 421.06 431.66 410.44 394.74 426.50 406.58 404.60 361.62 355.84 337.80 292.98 262.67 249.98 248.71 263.55 239.43 252.31 241.37 284.95 287.21 298.26 349.98 339.34 346.46 295.14 325.31 322.16 323.63 315.35 313.51 329.65 316.06

Weekly data were selected to avoid daily noise and focus on long-term trends and more stable seasonal cyclic. The study period covered approximately three years, providing a sufficient sample size for training, testing and encompassing a variety of market conditions (bullish, bearish, and stable). This dataset is characterized by its nonlinearity and complexity, making it an appropriate testing environment for assessing the hybrid model's ability to handle and predict complex temporal behavior. This data reflects dynamic changes in the financial market and provides a suitable environment for evaluating the model's ability to handle nonlinear and complex behaviors. This application aims to analyze the model's accuracy in predicting future observations. The program used for this analysis is MATLAB-R2022A.

**Step 1:** Enter the data. The given values represent a time series (Tesla Stock Price). They are entered into a single matrix on order to stabilize the model space and define the time period to ensure repeatability.

**Step 2:** Convert the series to a vertical vector. Convert the data into a vertical vector (ts) format for easier processing and analysis.

**Figure 3.**
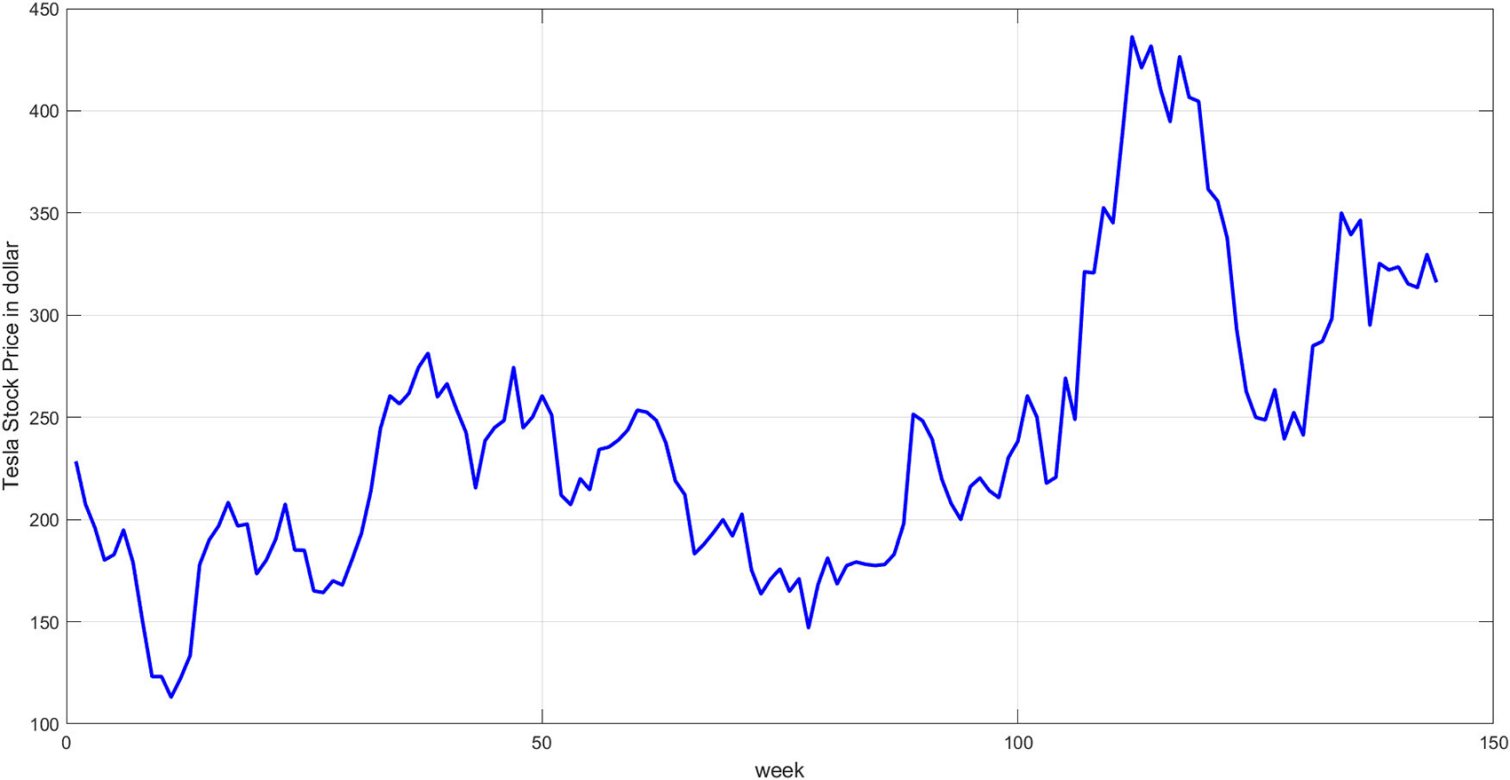
Plot of tesla stock price.

**Step 3:** Plot the original time series. Plot the original prices to provide an initial overview of the temporal behavior. Figure 3 illustrates the plot of the data series.

Figure 3 displays the original time series data for Tesla stock prices from October 2022 to July 2025. The timeline reveals clear fluctuations, from periods of sharp decline to gradual rise, followed by a significant increase in the latter half of the series. The nonlinear, cyclical, and random nature of this graph highlights the challenges of modeling using traditional methods and underscores the need for a hybrid approach to address the inherent complexity.

**Step 4:** In order to test the generalization ability and prevent information leakage, 134 observations were used for training and 10 for final compression.

**Step 5:** Decomposing the Time Series into

• Trend using a 12-period (week) moving average: This step extracts the general trend from the time series using the movmean(ts,12) instruction. This means calculating the 12-period (week) moving average. The goal is to remove short-term fluctuations from the series and reveal the long-term trend (rise, fall, or stability). A 12-period moving average will produce a smooth trend that reveals the pattern of growth or decline without the noise, as shown in Equation (4).
• Dispersion after removing the trend: This step isolates the random component (dispersion or noise). Dispersion represents irregular or exceptional changes that the trend, seasonality, errors, anomalies, or unexpected events cannot explain.
• Seasonal: Assuming the periodicity is the difference between the origin and the trend, after extracting the trend, we subtract the trend from the vertical data vector and then divide by the dispersion values, as shown in Equation (7), to extract the seasonality component. This isolates recurring cyclical changes in a series, such as monthly or seasonal variations, that appear and disappear periodically. Result: If the series has a recurring pattern (for example, every 12 weeks), it appears here.

These steps are helpful because they enable better analysis and interpretation of the series, as well as the construction of customized predictive models for each component using a neural network. They also help improve forecasting accuracy by predicting the entire series at once rather than predicting each point.

Decomposing series reduces complexity and assigns each component a different smoothing behavior. The trends across a 12-week moving average removes short-term noise and shows the slow structure according the trend equation.

**Figure 4.**
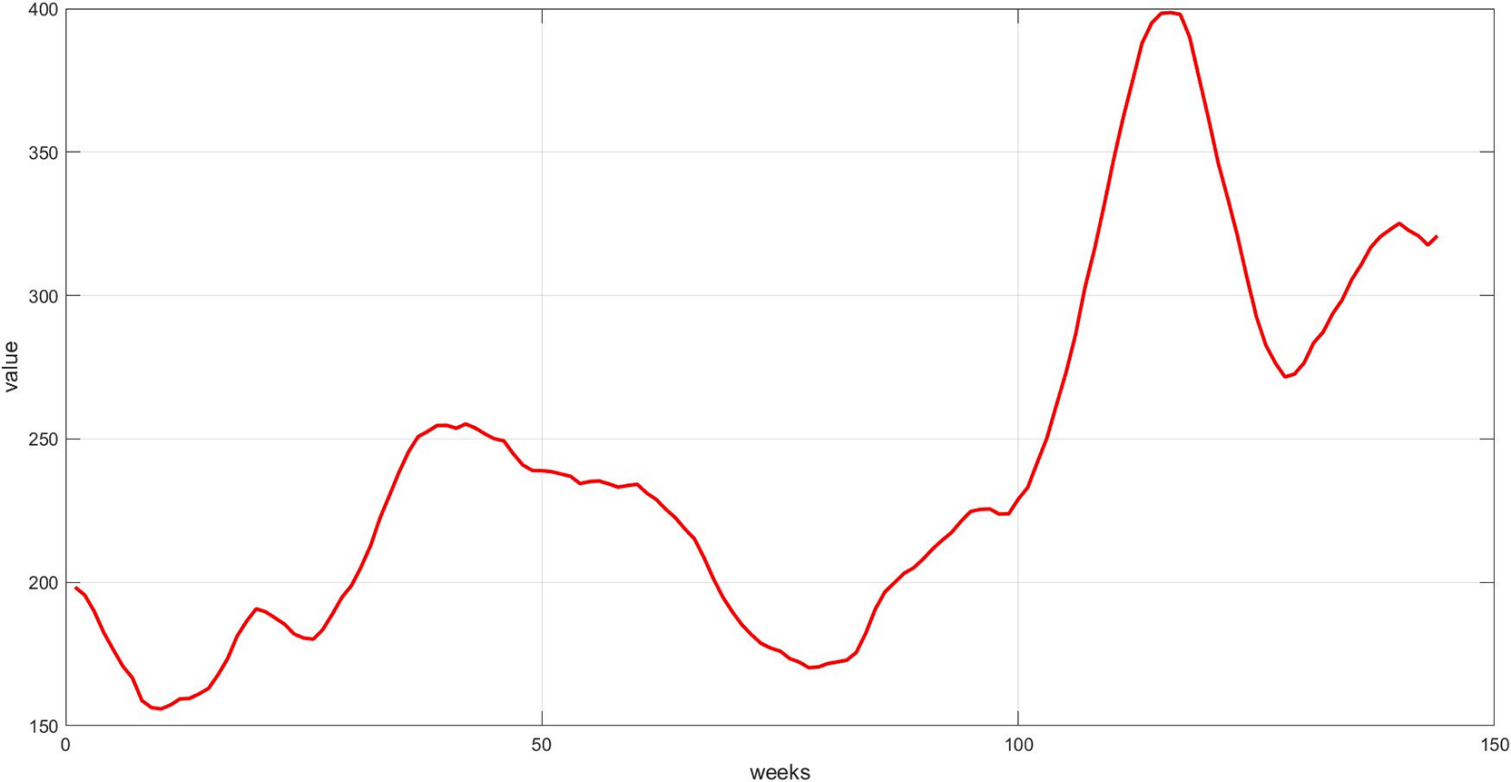
Plot the trend component for the training set.

**Figure 5.**
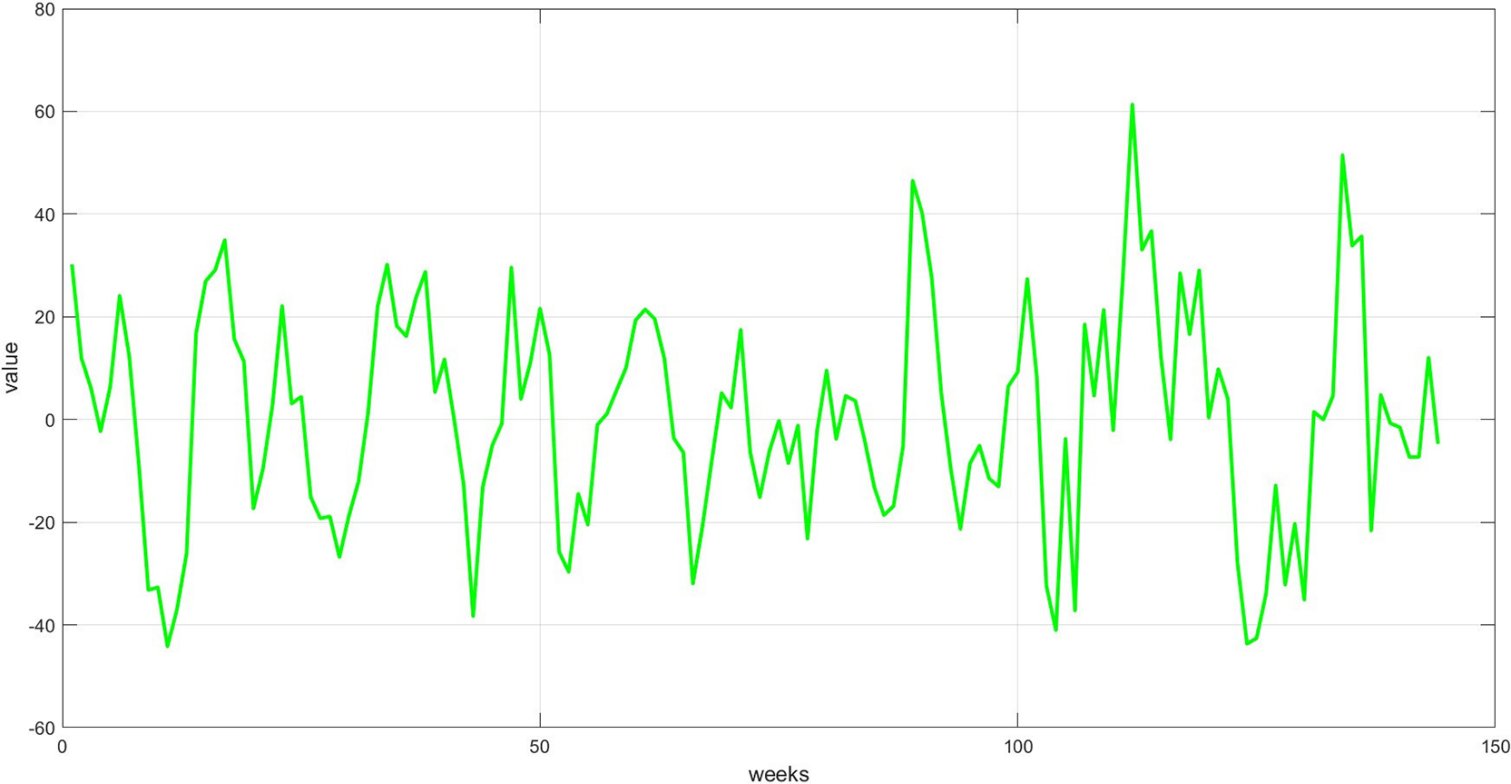
Plot the seasonal component for the training set.

**Figure 6.**
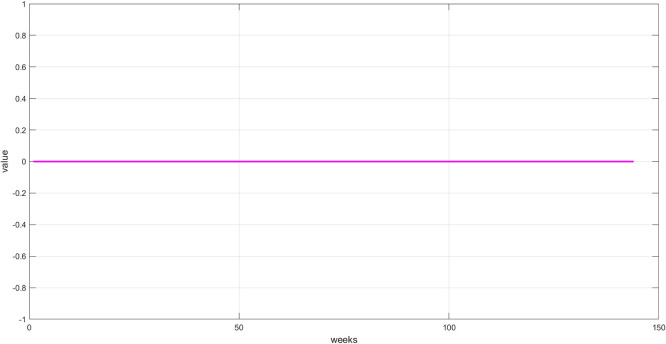
Plot dispersion component for the training set.

**Step 6:** Plot the three components. The trend, seasonality, and dispersion are plotted, as shown in Figures 4, 5, and 6.

Figure 4 represents the general long-term trend extracted via the 12-week moving average. The graph shows a smoothed path that captures the upward or downward trend in the series, after removing short-term noise. This graph is essential for understanding the structural growth of the series and guiding the model to handle fluctuations, rather than temporary variations.

Figure 5 depicts regular cyclical recurrences in the data, such as weekly variations. This component was derived by removing the trend and dividing the remainder by the dispersion component. The graph displays a recurring pattern, indicating a seasonal trend over a specific period, which the model can utilize to enhance forecasting.

Figure 6: This graph shows the unexplained random variations after removing both trend and seasonality. These values represent irregular fluctuations caused by market shocks or non-recurring factors. Their importance lies in containing residual signals that may contain crucial information the model must learn to accurately predict. The values appear to be zero, demonstrating the accuracy of the STD model's segmentation.

**Step 7:** Train neural networks for each component using RBFNN. It network is designed as a three- layer networks (input, hidden, and output). The connection type for each component in its independent, and the architecture is feedforward only, while the connection is fully connected between the hidden and output. There are no convolutional or feedback connections between three layers. As for the connection between input, hidden and output, there are no traditional weights; instead, vector functions are evaluated for each node and then fully connected to output. A moving average of 12 is used (12-week) is used to capture the trend. The number of layers for each network is three. The output layer is single node, as the input is the time index 1,…,N . The hidden layer has up to 97 nodes per RBF network, as in. ^1^ The network use fewer than 97 nodes if it reaches the error target of 0.001 before that the output layer has a single node, giving a single predication value for each time. the target value and width are set to 1, and maximum number of nodes adjusts the balance of bias and variance. RBFNN efficiently approximates nonlinear relationships with two-stage training of centers, then weights using a Gaussian function and a linear output formula, then calculating weights pseudo-inversely.

**Step 8:** Predict each component. Use SIM to predict each component of the time series separately.

**Figure 7.**
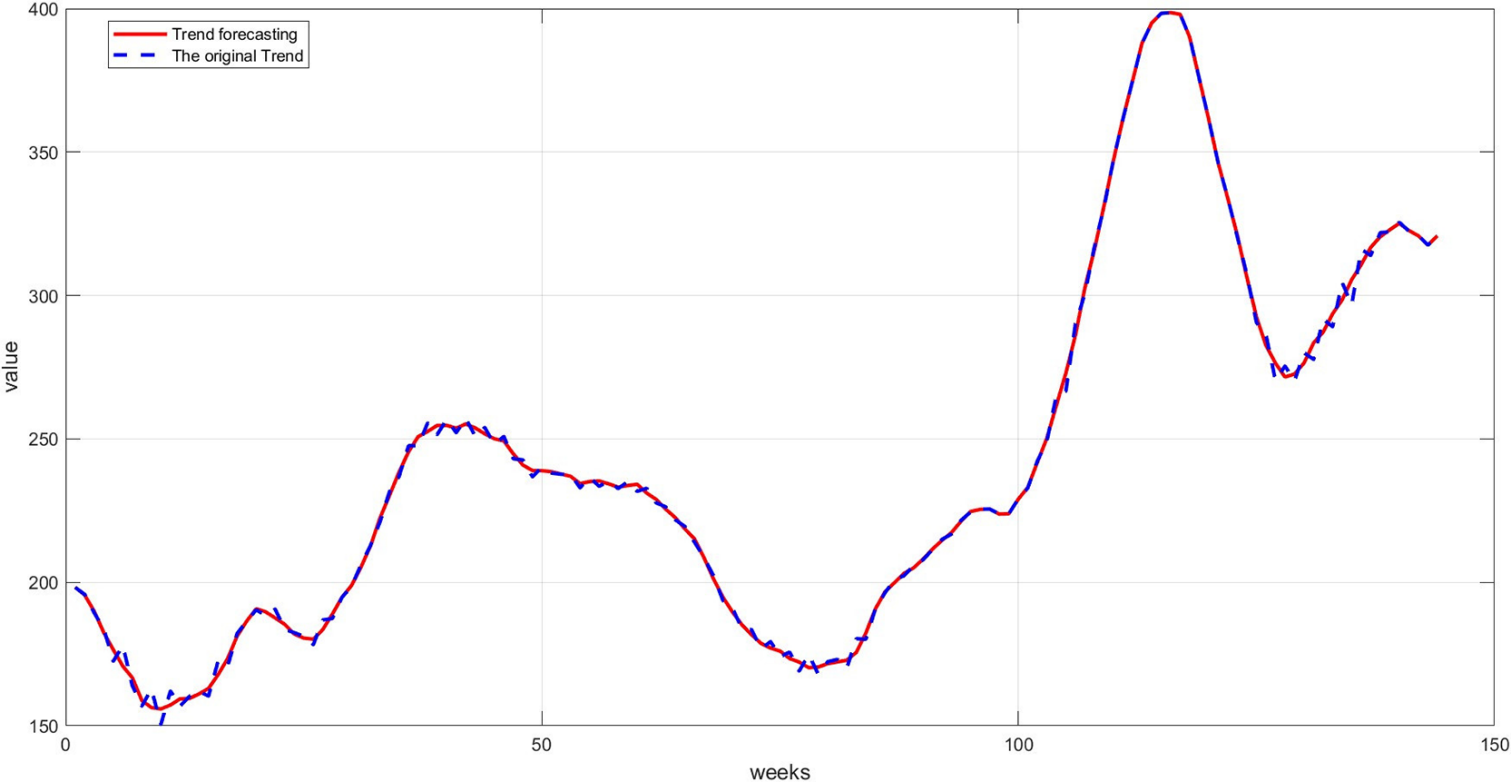
Plot trend: training and prediction.

**Figure 8.**
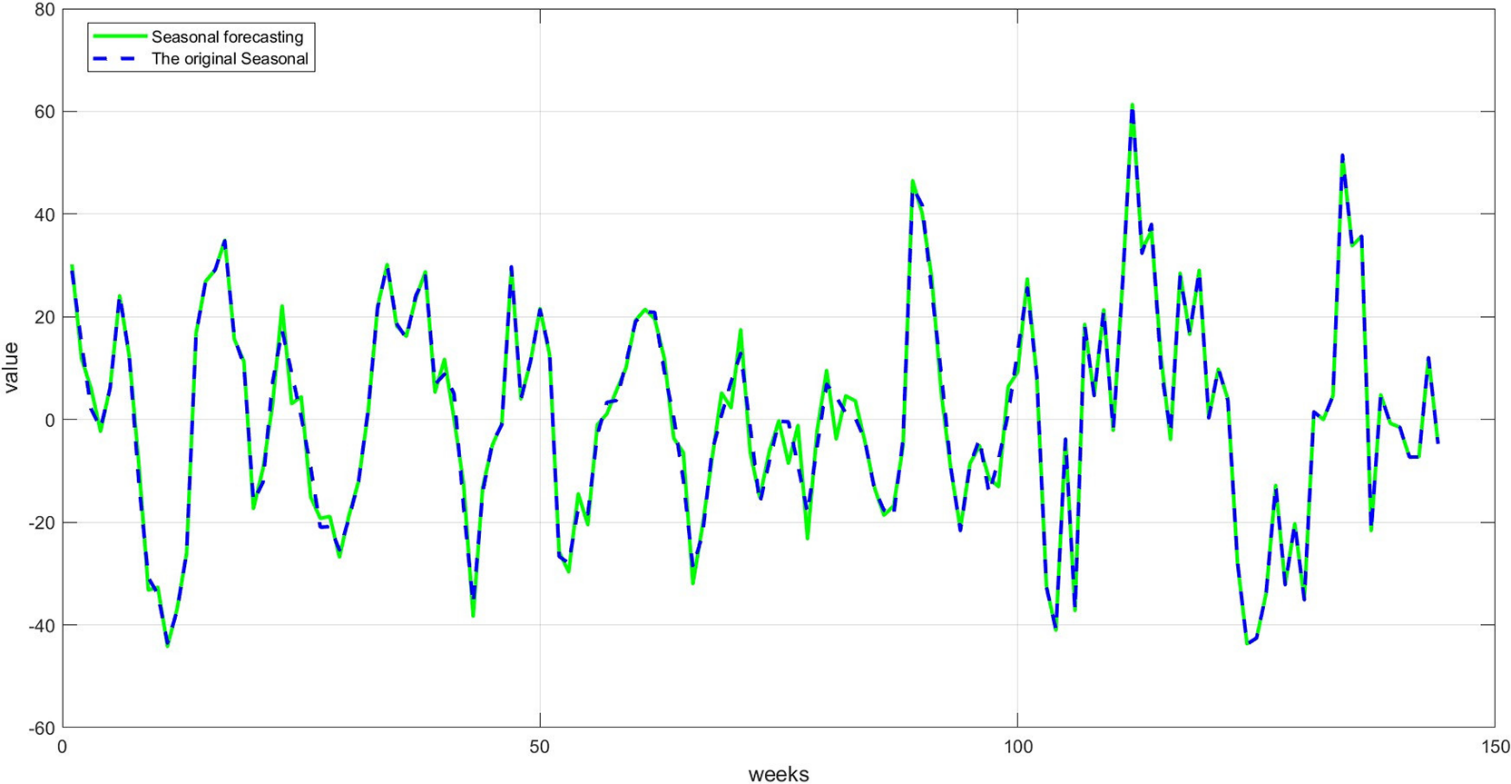
Plot seasonal: training and prediction.

**Figure 9.**
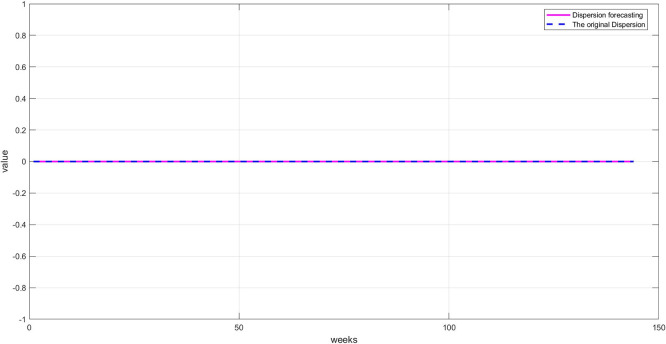
Plot dispersion: training and prediction.

**Step 9:** Plot the comparison between the original and predicted values for each component. The trend line, seasonality, and residuals are displayed next to the predicted values to assess the quality of the training, as shown in Figure 7 namely Plot Trend: Training and prediction, Figure 8 namely plot Seasonal: Training and prediction, and Figure 9 Plot Dispersion: Training and prediction.

Figure 7: This graph shows a comparison between the original trend component and the one predicted using RBFNN. We notice that the predictions are very close to the original values, demonstrating the model's ability to accurately learn the general trends. The deviation is slight, as reflected in the low mean squared error.

Figure 8: A comparison between the actual and predicted seasonal values. This graph demonstrates good replication of seasonal cycles, indicating the network's ability to learn this cyclical pattern. This performance enhances the reliability of the overall predictions when combining the components.

Figure 9: A good match between the original and predicted dispersion, with some expected differences due to the random nature of this component. However, the network was able to capture a large portion of these changes, reflecting the RBFNN's accuracy in processing nonlinear data.

**Step 10:** Reconstruct the predicted series by combining the three predicted components to reconstruct the complete time series.

**Figure 10.**
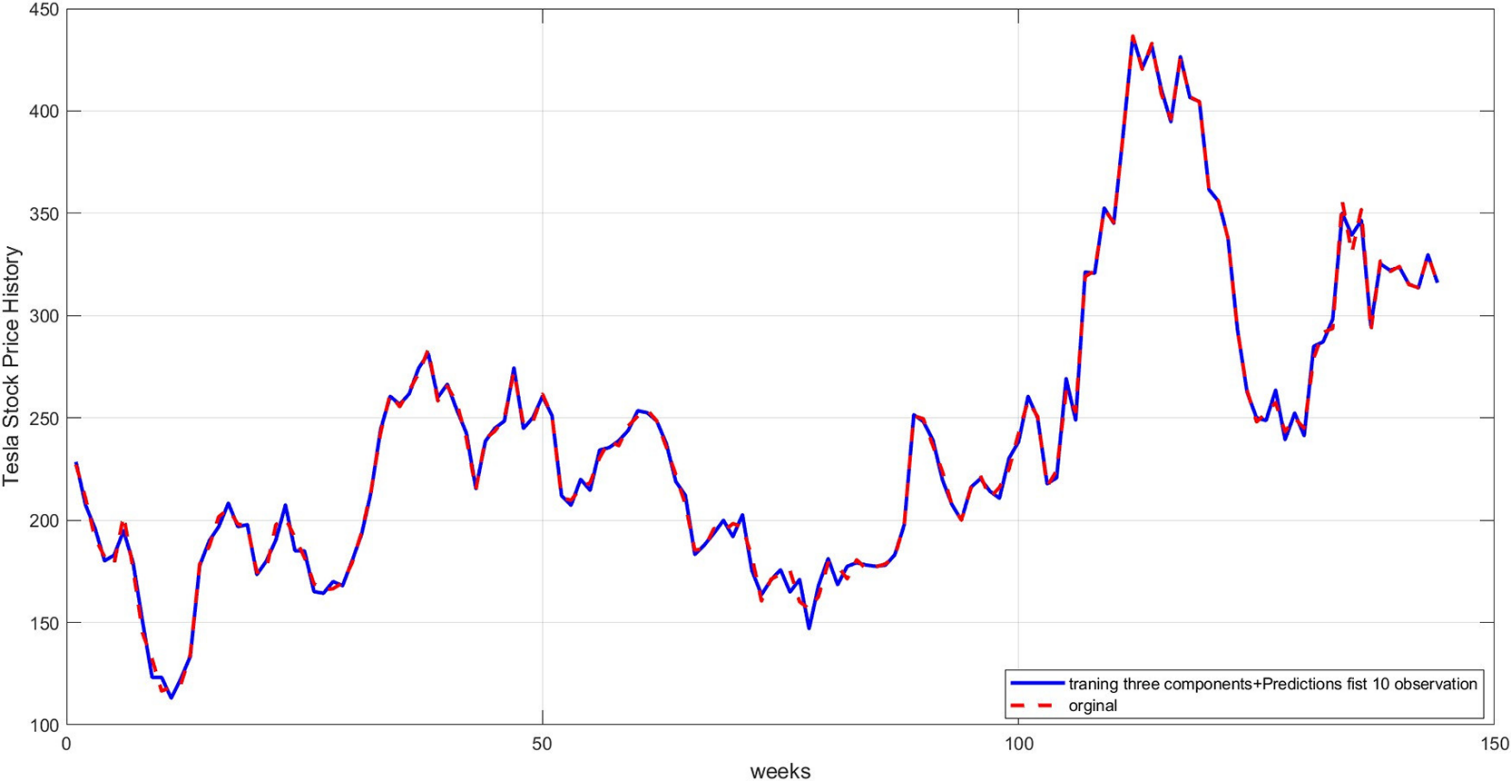
Plot of the original series and predictions.

**Step 11:** Plot the original series with the forecast. The original series is displayed alongside the predicted series to visually verify the model's accuracy, as shown in Figure 10.

Figure 10 illustrates a comparison between the actual time series and the final prediction obtained by combining the three components after training each component separately. The graph shows a good match, demonstrating the success of the proposed hybrid methodology in reconstructing the original series.

**Figure 11.**
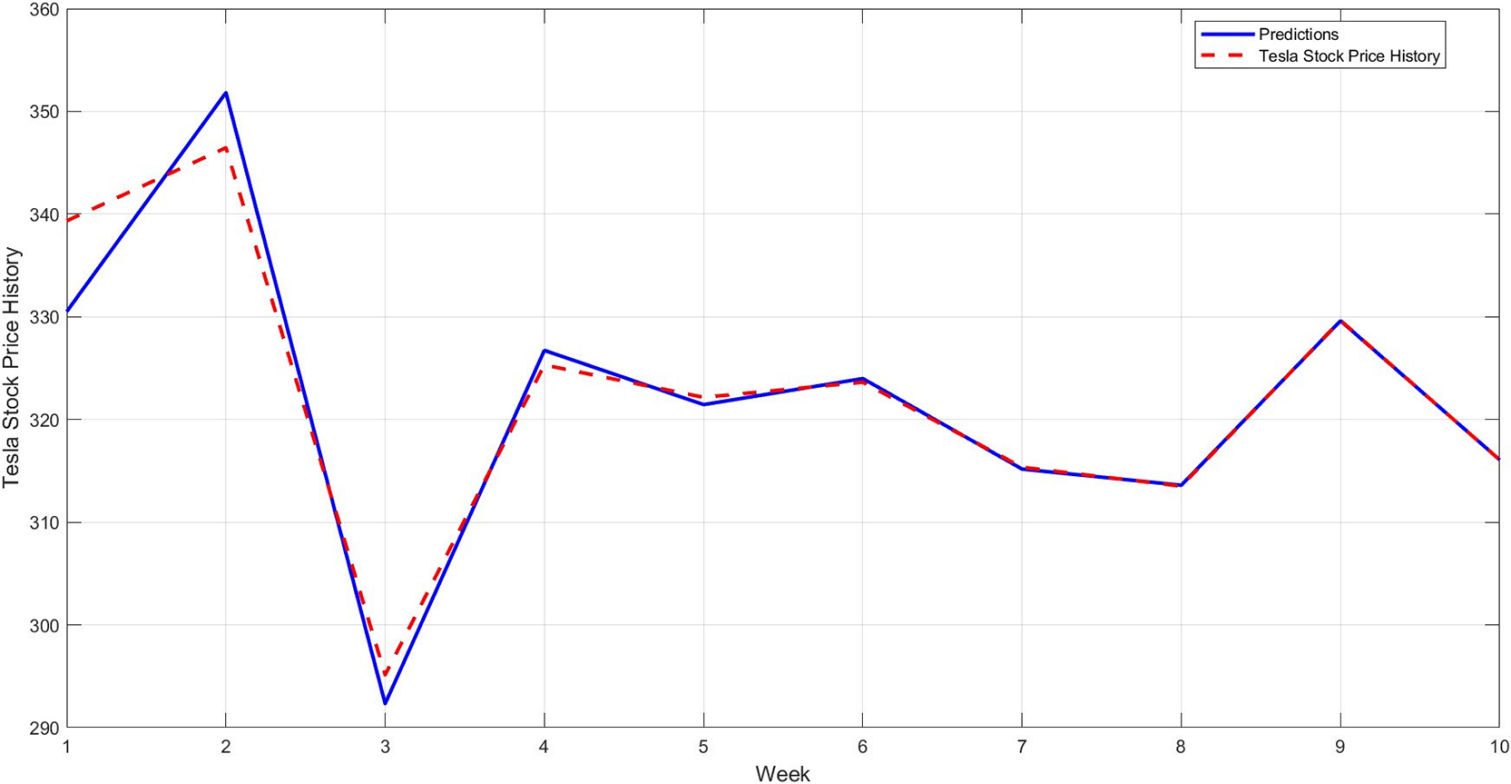
Comparison between original series and predictions by STD-RBFNN (last 10 observations).

**Figure 12.**
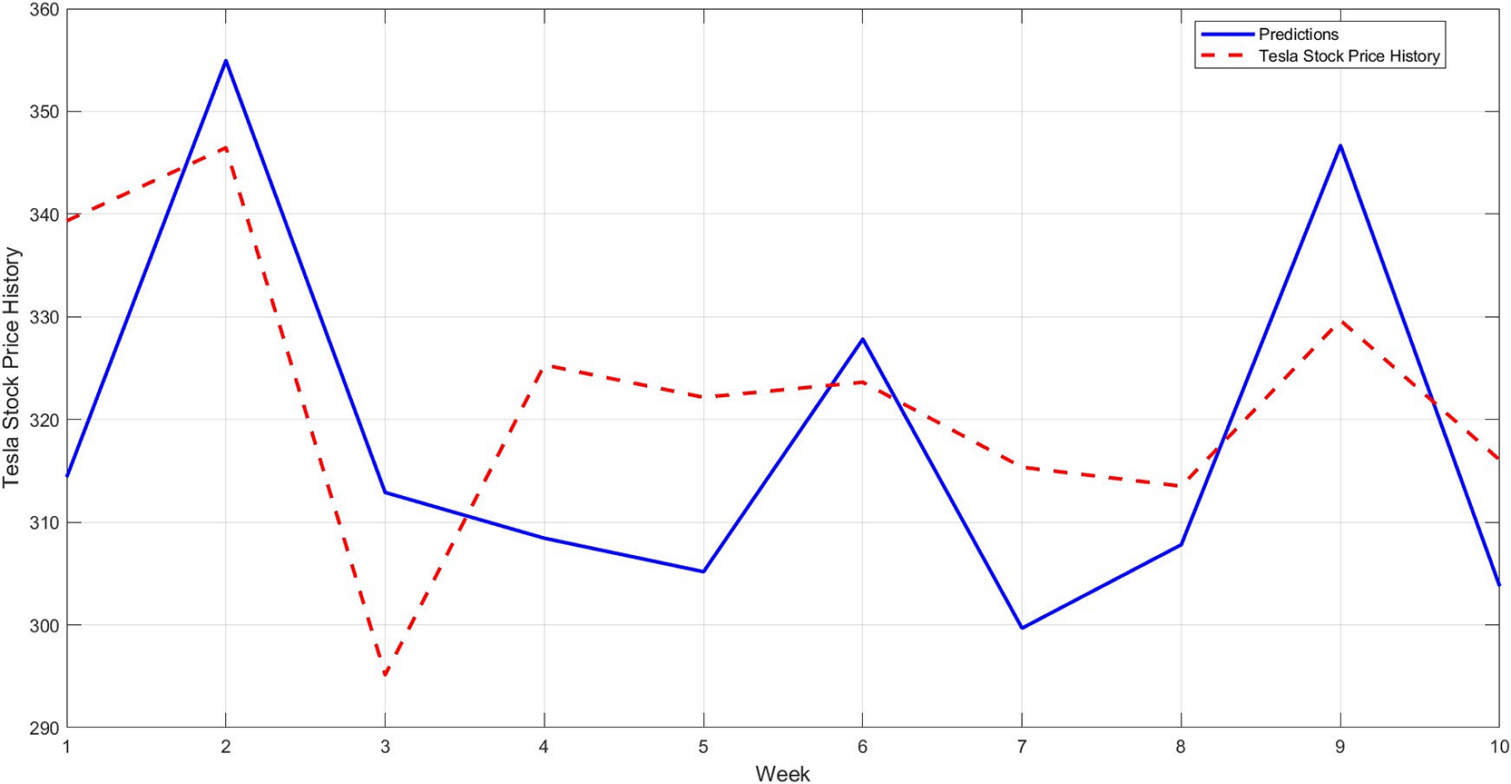
Comparison between original series and predictions by STRD-MNN (last 10 observations).

**Step 12:** Compare the predicted results with the actual values for the last 10 weeks, and plot the comparison between them, as shown in Figure 11. As a confirmation step to demonstrate the model's accuracy, a model is constructed using the STRD-MMN model, ^4^ Taking the same conditions as the proposed model, i.e. the number of hidden layers is up to 97 nodes for each network, the network may use a number less than 97 if it reaches the error target of 0.001. The comparison results for the last 10 observations are shown in Figure 12.

Figure 11 illustrates that the proposed model more accurately captures the actual price movement, with minimal deviation from the actual values. This reflects the model's ability to generalize to unseen data. Figure 12 shows another model (STRD-MNN) for comparison. A clear gap is evident between the predicted values and the original values, confirming that the new STD-RBFNN model is the most accurate and well-fitting model.

**Step 13:** Calculate the performance indicators for the improved hybrid STD-RBFNN and STRD-MMN models. The results are shown in Table 1 and are as follows:

**Table 1.** Comparison between the original series and predictions.

No.	Last 10 observations of the original series	Forecasting by STD-RBFNN	Forecasting by STRD-MNN
1	339.3400	**330.5043**	314.3918
2	346.4600	**351.8237**	354.9450
3	295.1400	**292.3326**	312.8973
4	325.3100	**326.7295**	308.44
5	322.1600	**321.4484**	305.1795
6	323.6300	**323.9857**	327.83
7	315.3500	**315.1728**	299.68
8	313.5100	**313.5972**	307.81
9	329.6500	**329.6091**	346.68
10	316.0600	**316.0753**	303.76
**MSE**		**11.7408**	**231.9654**
**RMSE**		**3.4265**	**15.2304**
**MAE**		**2.0117**	**13.9726**
**MAPE**		**0.6212%**	**4.4007%**
$R^{2}$		**0.9938**	**0.8775**

The STD-RBFNN prediction values show a very close match to true level at most points. The signed differences are small and balanced around zero. As examples are clear. At point 1, the prediction decreases by about 8.84. At point 2, it increases by about 5.36. After that, the differences become marginal: -2.81 at point3, 1.42 at point 4, -0.71 at point 5, 0.36 at point 6, -0.18 at point 7, 0.09 at point 8, -0.04 at point 9, and 0.02 at point 10. This pattern indicates the absence of systematic bias and stable tracking. Performance metrics confirm this. The MAE is only 2.01, MAPE is 0.62%, which is very low error level on a relative scale, RMSE is 3.43, meaning that large errors are rate. While $R^{2}$ is 0.9938 indicating that the model explains almost all of the variance in series at this point. Such a reading places STD-RBFNN as the first choice for short-term prediction for this series.

On the other hand, STRD-MNN exhibits large and more volatile errors. The dominant pattern trends to underestimate the true level at several points and then jump to overestimates at other points. At point 1, it underestimates by about 24.94. At point 2, it overestimates by 849. At point 3, it overestimates by 17.76. It then returns to a significant underestimate at points 4 and 5, at about -16.87 and -16.98. the underestimates are repeated at point 7 and 8, at about -15.67 and -5.70. the overestimates returns at point 9, at 17.03, and then underestimates by -12.30 at point 10. These fluctuations indicate increased sensitivity to trend component or to the way the outputs are reconstructed after decomposition. The figure summarize the situation. The MAE is 13.97, MAPE is 4.40%, RMSE is 15.23, and $R^{2}$ drops to 0.8775. These values reflect a wider spread of errors and more instability in tracking compared to first model.

## Conclusions

This work presents a hybrid time series forecasting STD-RBFNN framework. This decomposition addresses the heterogeneity of dynamics between components and reduces the complexity of learning the overall signal. The framework was applied to a weekly Tesla price series for the period from October 2022 to July 2025. A 12-week moving average was used to extract the trend. The three networks were trained, and their outputs were then combined to reconstruct the forecasted series. The STD-RBFNN model achieved superior performance compared to another hybrid framework, showing significantly higher error metrics on the same data and forecast horizon, supporting superiority and limiting sources of bias. This accuracy is attributed to the separation of learning by component and the Gaussian properties of RBF approximation of local nonlinearities. Adjusting the number of layers contributed to achieving a practical balance between bias and variance. The results indicate the transferability of the approach to financial, energy, and environmental series with similar seasonal and trend structures, while maintaining simplicity of implementation and interpretability through decomposition.

Challenges and limitations of the study include univariate modeling, the assumption of constant seasonality, and the failure to test for significant structural shocks or time-varying seasonality. The study recommends extending the framework to multivariate models, experimenting with alternative radial functions and self-regulating the number of nodes and width, adopting multi-window rolling estimations, and estimating confidence intervals for forecasts to ensure higher robustness and better reproducibility.

## Data Availability

Zenodo. Tesla stock prices (data). https://doi.org/10.5281/zenodo.18343726 (Noori, 2026). This project contains the following underlying data: Tesla_stock_prices.xlsx (Weekly closing prices of Tesla stock used for all empirical analyses, model estimation, and forecasting procedures reported in the study.)
