# Supplementary material for: The Improved Hybrid STD– Radial Basis Function Neural Network Approach for Time Series Forecasting Application to Tesla Stock Price Prediction
Source: F1000Res. 2026 Mar 28;15:286. Originally published 2026 Feb 18. [Version 2] doi: 10.12688/f1000research.172498.2 (PMC13070210; doi:10.12688/f1000research.172498.2)
Supplement: Peer review history

# Reviewer report: Reviewer response for version 2

**DOI:** 10.5256/f1000research.197569.r471354

**Published:** 2026-04-11

**Authors:** John Kamwele Mutinda

**Affiliations:** 1 African Institute for Mathematical Sciences, Cape Town, South Africa

I would like to acknowledge the authors’ efforts in revising the manuscript. While not all of my previous comments have been fully addressed, the revisions have resulted in a noticeably clearer and more readable paper compared to the earlier version.

 The methodological presentation of the STD–RBFNN forecasting framework has been improved, particularly regarding the decomposition and prediction steps. The mathematical notation is now more consistent and easier to follow, which enhances the technical transparency of the work. Additionally, the benchmark comparison is presented more clearly, allowing readers to better assess the relative performance of the proposed approach. The discussion section has also been revised to more accurately reflect the scope and limitations of the case study, which adds appropriate nuance to the claims.

 Several language and consistency edits throughout the manuscript further contribute to its overall coherence. While some concerns remain outstanding, the current version represents a step in the right direction. The authors have made a genuine attempt to improve clarity, and the manuscript is now in a stronger position to convey its core contributions.

Is the work clearly and accurately presented and does it cite the current literature?

No

If applicable, is the statistical analysis and its interpretation appropriate?

Partly

Are all the source data underlying the results available to ensure full reproducibility?

Yes

Is the study design appropriate and is the work technically sound?

No

Are the conclusions drawn adequately supported by the results?

No

Are sufficient details of methods and analysis provided to allow replication by others?

Partly

Reviewer Expertise:

Time series forecasting, deep learning and machine learning

I confirm that I have read this submission and believe that I have an appropriate level of expertise to confirm that it is of an acceptable scientific standard.

# Reviewer report: Reviewer response for version 1

**DOI:** 10.5256/f1000research.190231.r460209

**Published:** 2026-03-06

**Authors:** John Kamwele Mutinda

**Affiliations:** 1 African Institute for Mathematical Sciences, Cape Town, South Africa

\section{Overall Assessment and Recommendation}

 This manuscript proposes a hybrid model combining seasonal-trend decomposition with a radial basis function neural network, applied to weekly Tesla stock price prediction. The approach decomposes the series into trend, seasonal, and residual components, models each using RBFNN with Gaussian basis functions, and recombines the forecasts. While the general concept of decomposition-based hybrids is reasonable, the work suffers from major shortcomings in novelty, methodological depth, scope, benchmarking, statistical validation, and presentation. 

 \section{Innovation and Novelty}

 The study lacks sufficient novelty. Hybrid models that integrate classical decomposition techniques with neural network predictors have been extensively explored in the financial time series forecasting literature. The specific pairing of seasonal-trend decomposition and RBFNN does not constitute a significant methodological advance. The authors must clearly explain what makes their contribution distinct from existing decomposition-based hybrid approaches.

 \section{Introduction}

 The introduction is brief and lacks sufficient depth. It fails to adequately explain the theoretical motivation for hybrid models or why they are needed over standalone classical machine learning or deep learning methods. The research gap is not convincingly established, and the proposed model is not well positioned within the current state of the art.

 \section{Literature Review}

 The literature review is limited and insufficiently comprehensive. Recent research has demonstrated the effectiveness of hybrid models that integrate signal processing for preprocessing with advanced forecasting architectures such as transformers and informers, which have shown strong performance in financial time series prediction. The authors should expand this section and include a summary table classifying prior studies by dataset, time period, methods used, main forecasting model, and key contributions. This would better position the current work and highlight its unique contribution.

 \section{Data and Methods}

 The data source, time span, and preprocessing steps, including handling of missing values, are not clearly described. The rescaling method and whether it was applied to the entire dataset or separately after train-test splitting to prevent temporal leakage should be explicitly stated.

 The evaluation relies on a single hold-out train-test split without time-series-aware cross-validation or multiple hold-out partitions, limiting the assessment of generalization ability. The study uses only one stock, Tesla, which severely restricts generalizability. Including at least three to five additional stocks would strengthen the results.

 The benchmark set is narrow. Classical statistical models such as ARIMA and ARMA, advanced deep learning architectures such as TCN, transformers, informers, and CNN, and other recent hybrid models from the literature are absent. This weakens the claim of superiority.

 Strict statistical robustness checks, including Diebold-Mariano, modified Diebold-Mariano, and Model Confidence Set tests, should be performed to confirm statistical significance. Visual comparison of performance metrics using bar plots, heatmaps, or line graphs would improve interpretability.

 \section{Discussion}

 The discussion is primarily empirical and lacks depth. It does not sufficiently explore the internal mechanisms behind the observed predictive performance. A rigorous explanation of why the decomposition step and RBFNN modeling lead to improved accuracy is needed.

 \section{Conclusion}

 The conclusion should clearly restate how the results align with the stated objectives, explicitly acknowledge limitations, and outline concrete directions for future research.

 \section{Language and Presentation}

 The manuscript contains numerous grammatical errors and typos that impair readability and require careful proofreading.

Is the work clearly and accurately presented and does it cite the current literature?

No

If applicable, is the statistical analysis and its interpretation appropriate?

Partly

Are all the source data underlying the results available to ensure full reproducibility?

Yes

Is the study design appropriate and is the work technically sound?

No

Are the conclusions drawn adequately supported by the results?

No

Are sufficient details of methods and analysis provided to allow replication by others?

Partly

Reviewer Expertise:

Time series forecasting, deep learning and machine learning

I confirm that I have read this submission and believe that I have an appropriate level of expertise to state that I do not consider it to be of an acceptable scientific standard, for reasons outlined above.

# Response

**Published:** 2026-03-09

**Authors:** Nooraldeen Alsaab

**Affiliations:** Mathematics, Anbar General Directorate of Education, Ministry of Education, Fallujah, Iraq

We sincerely thank the reviewer for the careful reading and constructive comments. We have revised the manuscript extensively in response to the concerns raised. In the revised version, we clarified the actual contribution of the study, strengthened the Introduction and theoretical motivation for the hybrid framework, improved the description of the dataset and empirical design, added a separate Discussion section, revised the Conclusion to better reflect the actual scope of the results, and corrected numerous language and presentation issues throughout the manuscript. We also moderated claims of novelty and generalizability and explicitly acknowledged the current limitations of the study.

**Regarding novelty**, the manuscript has been revised to present the contribution more precisely. The study is now framed as the development and empirical evaluation of a structured STD–RBFNN forecasting framework for a weekly univariate stock series, rather than as a wholly new forecasting paradigm.

**Regarding the Introduction and literature positioning**, we expanded the theoretical motivation for hybrid forecasting and clarified why decomposition-based learning may be beneficial for complex financial series. The research gap has been rewritten more carefully to better position the study within the existing literature.

**Regarding the data and methods**, we improved the description of the Tesla dataset, clarified the study period and number of observations, and revised the explanation of the chronological train–test split. We also made the empirical setting more transparent and explicitly acknowledged that the current design is based on a single hold-out evaluation.

**Regarding benchmarking and generalizability**, we agree that the empirical comparison remains limited. We therefore revised the manuscript to avoid broad superiority claims and clearly stated that the results should be interpreted within a restricted benchmark and single-asset setting. This limitation is now explicitly discussed in the manuscript.

**Regarding statistical robustness**, we acknowledge that formal procedures such as Diebold–Mariano, modified Diebold–Mariano, and Model Confidence Set tests were not included in the present version. This limitation is now clearly stated, and these tests are proposed as part of future work.

**Regarding the Discussion and Conclusion**, we added a clearer explanation of why decomposition followed by component-wise RBFNN modeling may improve forecasting accuracy. We also revised the conclusion to align more closely with the study objectives, explicitly state the limitations, and outline concrete directions for future research.

**Regarding language and presentation**, the manuscript has been carefully proofread and revised to improve clarity, grammar, and overall readability.

 We are grateful for the reviewer’s comments, which helped us improve the manuscript substantially.

# Reviewer report: Reviewer response for version 1

**DOI:** 10.5256/f1000research.190231.r460200

**Published:** 2026-03-05

**Authors:** Yogeesh N

**Affiliations:** 1 Government First Grade College, Tumkur, Karnataka, India

This article presents a clear and well-structured hybrid forecasting framework that combines Seasonal-Trend-Dispersion decomposition with Radial Basis Function Neural Networks for financial time-series prediction. The study is motivated well in the introduction, where the authors position the work within the broader literature on hybrid forecasting models and identify the specific novelty of integrating STD decomposition with RBFNN architecture for stock-price prediction. The rationale for decomposing the original series into trend, seasonality, and dispersion before nonlinear modeling is coherent and appropriately supported by prior work cited in the manuscript.

 The methodology is presented in a logically sequenced manner. The paper first defines the STD decomposition mathematically, then explains the RBFNN architecture and training process, and finally integrates both into the proposed STD-RBFNN pipeline. The workflow is sufficiently detailed, including decomposition, train-test splitting, component-wise RBFNN training, recombination of predicted components, and evaluation using standard forecasting metrics such as MSE, RMSE, MAE, MAPE, and R². The application section also states the data period, the use of weekly Tesla stock prices, the use of a 12-week moving average, the training/test split, and the MATLAB environment used in implementation.

 The study design is appropriate for the stated objective. The authors use a real-world financial dataset with nonlinearity and volatility, which is a relevant and demanding test bed for the proposed model. The decision to model the decomposed components separately is technically sound and aligns with the underlying premise that different components of a financial time series may exhibit different dynamics. The holdout evaluation on the last 10 observations and the direct comparison against an alternative hybrid model provide a reasonable demonstration of comparative predictive performance.

 The reported results are strong and support the main claims of the paper. On the last 10 observations, the proposed STD-RBFNN model achieves markedly lower error metrics than the comparison STRD-MNN model, with MSE of 11.7408 versus 231.9654, RMSE of 3.4265 versus 15.2304, MAE of 2.0117 versus 13.9726, MAPE of 0.6212% versus 4.4007%, and R² of 0.9938 versus 0.8775. These results convincingly indicate that the proposed model tracks the short-horizon movement of the Tesla series more closely under the experimental conditions used in the manuscript.

 I also note positively that the authors have made the underlying dataset available through Zenodo, which materially strengthens reproducibility and transparency. The manuscript explicitly states that the uploaded Excel file contains the weekly closing prices used for empirical analyses, model estimation, and forecasting.

 Overall, this is a useful and publishable contribution. The manuscript is readable, the proposed hybrid framework is motivated and technically justified, and the empirical comparison shows clear gains over the benchmark model included by the authors. The conclusions are consistent with the numerical results and are appropriately framed, while still acknowledging limitations such as univariate modeling, constant seasonality assumptions, and the absence of explicit testing for structural shocks or time-varying seasonality.

 Minor comments for optional improvement

 These points are not major criticisms and do not affect the scientific soundness of the work, but addressing them would further improve the paper:

1. The manuscript would benefit from a light language edit for grammar and phrasing in a few sections.
2. A final consistency check of terminology would help, especially where “STD-RBFNN” and “STL-RBFNN” appear in nearby sections.
3. The paper could be strengthened further by briefly stating whether any sensitivity analysis was performed for the number of hidden nodes and width parameters, although the current presentation is already adequate for understanding the workflow.

Is the work clearly and accurately presented and does it cite the current literature?

Yes

If applicable, is the statistical analysis and its interpretation appropriate?

Yes

Are all the source data underlying the results available to ensure full reproducibility?

Yes

Is the study design appropriate and is the work technically sound?

Yes

Are the conclusions drawn adequately supported by the results?

Yes

Are sufficient details of methods and analysis provided to allow replication by others?

Yes

Reviewer Expertise:

AI, Mathematical Education, Numerical Technique, Modeling

I confirm that I have read this submission and believe that I have an appropriate level of expertise to confirm that it is of an acceptable scientific standard.

# Response

**Published:** 2026-03-09

**Authors:** Nooraldeen Alsaab

**Affiliations:** Mathematics, Anbar General Directorate of Education, Ministry of Education, Fallujah, Iraq

We sincerely thank the reviewer for the careful evaluation of our manuscript and for the constructive and encouraging comments. The feedback helped us improve the clarity and consistency of the paper. All suggested improvements have been carefully addressed in the revised version of the manuscript. The main revisions are summarized below.

 First, the reviewer suggested checking the consistency of terminology where “STD-RBFNN” and “STL-RBFNN” appeared in nearby sections. We carefully reviewed the entire manuscript and corrected all occurrences to ensure consistent use of the term “STD-RBFNN”, which accurately reflects the Seasonal-Trend-Dispersion decomposition used in this study.

 Second, the reviewer recommended briefly clarifying whether sensitivity analysis was conducted for the neural network parameters, particularly the number of hidden neurons and the width parameter of the radial basis functions. In response, we added a short explanatory paragraph in the RBF neural network methodology section. This paragraph explains that preliminary exploratory experiments were conducted to determine appropriate values for these hyperparameters, and that the final configuration was selected based on validation error stability.

 Third, following the reviewer’s suggestion, we performed a light language revision to improve clarity and grammatical consistency throughout the manuscript.

 We believe that these revisions have improved the clarity and presentation of the study while preserving the original methodology and results. We are grateful for the reviewer’s positive assessment of the work and for recognizing its contribution to hybrid time-series forecasting models.
